# Reported Changes in Dietary Habits During the COVID-19 Lockdown in the Danish Population: The Danish COVIDiet Study

**DOI:** 10.3389/fnut.2020.592112

**Published:** 2020-12-08

**Authors:** Davide Giacalone, Michael Bom Frøst, Celia Rodríguez-Pérez

**Affiliations:** ^1^Department of Technology and Innovation, University of Southern Denmark, Odense, Denmark; ^2^Department of Food Science, University of Copenhagen, Frederiksberg, Denmark; ^3^Department of Nutrition and Food Science, Faculty of Health Sciences of Melilla, University of Granada, Melilla, Spain; ^4^Biomedical Research Centre, Institute of Nutrition and Food Technology (INYTA) ‘José Mataix’, University of Granada, Granada, Spain

**Keywords:** COVID-19, lockdown, dietary habits, public health nutrition, Denmark

## Abstract

This paper focuses on the effect of the COVID-19 lockdown on the dietary habits of adult Danes. Two aspects were specifically considered: 1) reported changes in intake of specific food categories and 2) effect on healthy eating, operationalized as adherence to the Mediterranean diet (MEDAS score). Respondents (*N* = 2,462) completed a 44-items self-administered online survey designed for the assessment of their socio-demographic characteristics, general food habits, and consumption frequency of selected foods (mainly related to the MedDiet) during the lockdown. The data indicated that the lockdown has affected dietary habits of adult Danes to a relatively limited degree. The most important findings were that a substantial proportion of respondents (≥28%) reported eating more, snacking more, exercising less, and gaining weight during the lockdown. Results could be linked to the amount of time spent at home (e.g., a higher cooking frequency) a higher degree of emotional eating during the lockdown (e.g., a higher consumption of pastries and alcohol). Women were generally affected to a higher degree than men. Additionally, dietary changes during the lockdown to a certain degree reflected pre-existing (un)healthy eating habits, as positive health outcomes were observed in respondents with a high MEDAS score and negative outcomes (e.g., weight gain and higher intakes of pastries and carbonated beverages) were associated with respondents with a low MEDAS score. These changes, if sustained long-term, are potentially concerning from a public health perspective, especially given that more than half of the respondents were characterized by a low adherence to the MedDiet.

## Introduction

### Impact of COVID-19 on Dietary Habits—The COVIDiet Project

The 2019 coronavirus (COVID-19) pandemic, caused by SARS-CoV-2, has expanded from Wuhan, China to a growing number of countries (1). At the time of writing (October 21, 2020), more than 40 million confirmed cases of COVID-19 have been reported worldwide, of which about 5.2 million were in Europe according to the European Centre for Disease Prevention and Control (2).

In an effort to mitigate the spread of the pandemic, most European countries implemented a variety of measures limiting their citizens' freedom of movement (e.g., social distancing, ban on travel and public gathering) and mandated a lockdown of most societal activities (3). These extraordinary measures understandably caused significant disruption to most people's routines and lifestyle, and are expected to have exerted significant effects on dietary habits and physical activity (4, 5). For instance, as people spent much more time at home than usual, they had more time for cooking, as well as for snacking (5); restrictions to freedom of movement may have impacted food provisioning practices as well as access and availability for specific food items (6); boredom, stress and anxiety associated with the lockdown measures (7) likely increased emotional eating (8), and so on.

In this context, the COVIDiet_INT project has been established to estimate the impact of COVID-19 related lockdown measures on eating habits among the adult population. COVIDiet INT (9) is an international, crowd-sourced online study translated in 16 languages and conducted in 19 European countries, as well as four non-European countries (Colombia, Egypt, India, and Kuwait). Cross-sectional data from this project have recently been presented with respect to the effect on the Spanish population (5), as well as in a comparative paper (Rodríguez-Pérez et al., submitted).

These results indicate some reason for concerns, particularly as many respondents reportedly ate more, gained weight, and exercised less, due to limitation in outdoors and in-gym physical activity. However, the results also indicated a higher adherence to the Mediterranean Diet (MedDiet) during the lockdown, including higher intake of fruits, vegetables or legumes and lower intake of red meat, alcohol, fried foods, or pastries compared to people's usual habits (5). Since the MedDiet is regarded as the standard for healthy nutrition (10) this result could be seen as a positive outcome that, if it can be sustained in the long term, could help prevent the onset of chronic diseases and COVID-19 related complications.

It remains to be seen whether these effects extend to other countries, given the existence of local difference in dietary habits and in the severity of the lockdown measures. Therefore, this brief report, situated within the COVIDiet international project, seeks to replicate these earlier results and to provide a more detailed characterization of the effect of the lockdown measures on the Danish adult population.

### Brief Description of the Lockdown Measures in Denmark

This section provides a brief description of the lockdown measures undertaken by the Danish government. It is mainly based on publicly available information provided by the Danish Health Authority (“Sundhedstyrelse,” https://www.sst.dk/en/English). Denmark was among the first European countries to introduce lockdown measures, following similar measures introduced in countries such as China and Italy. While a number of recommendations were already issued in late February/early March, the first actual lockdown measures were officially declared on March 13, 2020, when all people working in non-essential functions in the public sector were ordered to stay home for two weeks. Concurrently, authorities urged employers in the private sector to allow their employees to work from home wherever possible (with the exception of workers in essential functions, such as, e.g., pharmacies, food retailers, and maintenance of critical infrastructure). Also, on 13 March, all secondary education institutions, universities, libraries, indoor cultural institutions and similar places were closed, initially for 2 weeks. Primary schools and daycare facilities were also closed down soon thereafter (March 16, 2020), with virtual (online) schooling used to some degree as a replacement. Further restrictions were implemented on March 18th: specifically, it became illegal to assemble more than 10 people in public, all shopping centers and stores involving close human contacts (e.g., hairdressers and nightclubs) were closed down, and restaurants could be open but only for take-away. On 23 March, the authorities announced that all lockdown measures would be extended and remain in place until 13 April. By late March, lockdown measures proved largely successful in mitigating the number of new cases and were gradually rolled back from mid-April. Specifically, nurseries, kindergartens and primary schools opened again on 15 April, whereas a broader reopening of activities took place on 11 May (shops) and 18 May (schools from 6th grade on, cafés, restaurants, hairdresser).

At the time of writing (October 21, 2020) Denmark has had more than 37,000 confirmed COVID-19 cases (11).

### Research Objectives and Study Design

As previously mentioned, this study is part of a larger effort to study changes in dietary behavior in the adult population (5). This specific paper presents data obtained from a Danish cohort and mainly focuses on two research objectives: (1) to report changes in the intake of specific food categories during the lockdown and (2) estimating the effect of lockdown on adherence to MedDiet. To this end, a self-administered web-based questionnaire was carried out with questions aimed at assessing the dietary behaviors of the adult Danish population during the lockdown period. The results from the survey are also compared with reports from companies, media and public institutions regarding observed changes in purchase behavior etc., to ascertain whether the observed impact of the reported changes are generalizable.

## Methods

### Measures

A 44-item self-administered online questionnaire was designed for the assessment of the respondent's socio-demographic characteristics, general food habits, and consumption frequency of selected foods (mainly related to the MedDiet). The selected foods included 14 items with reference to the MedDiet pattern based on the validated PREDIMED MedDiet Adherence Screener (MEDAS), whose score ranges from 0 to 14 points (12); for each item, participants were asked whether they had made any actual change due to the lockdown. Participants were also asked to answer 21 in-house items aimed at investigating changes in their general dietary habits during the lockdown, including frequency of cooking and snacking, alcohol intake, cooking methods, among others. All questions were designed to gauge whether participants increased, decreased or maintained their habits during the lockdown period. Additionally, participants were also asked whether their physical activity and body weight had changed since the lockdown started.

The full survey is available online at https://www.mdpi.com/2072-6643/12/6/1730/s1 (the Danish translation can be obtained from the authors upon request). We further refer to Rodríguez-Pérez et al. (5) and to the COVIDiet registered trial on clinicaltrials.gov (Identifier: NCT04449731) for a detailed walkthrough of the survey items. The study was approved by the Research Ethics Committee of the University of Granada (1526/CEIH/2020).

### Participants

Participants—reached using various channels, including internal databases, word of mouth and social media—completed the COVIDiet survey during the lockdown period (from 24 April to 5 May). There were no specific inclusion or exclusion criteria except that participants had to be of at least 18 years of age in order to give their informed consent. Respondents received no monetary incentive for their participation. A detailed breakdown of the characteristics of the sample population is given in Table 1.

**Table 1:** Socio-demographic and anthropometric characteristics of the sample population (*N* = 2,462).

**Background variable**	***N* = 2,462**	**%**
**Gender**		
Men	708	28.7
Women	1,750	71.1
Other	4	0.2
**Age**		
18-35	870	35.3
36-50	917	37.2
51-65	578	23.5
65+	97	3.9
**BMI (Mean** **=** **24.6 kg/m**^**2**^**, SD** **=** **4.4)**		
Underweight (<18.5)	48	2.0
Normal weight (18.5–24.9)	1,473	59.8
Overweight (25–29.9)	691	28.1
Obese (≥30)	249	10.1
**MEDAS (Mean** **=** **5.4, SD** **=** **2.1)**		
Low (≤5)	1,250	50.8
Medium (6-8)	1,043	42.4
High (≥9)	169	6.9
**Education**		
Higher education (Bachelor and above)	2,180	88.5
Medium ed. (High school and vocational training)	266	10.8
Basic education	7	0.3
Other	9	0.4
**Children in care**		
Yes	1,259	51.1
No	1,203	48.9
**Region**		
Hovedstaden	841	34.2
Midtjylland	130	5.3
Nordjylland	21	0.9
Sjælland	126	5.1
Syddanmark	1,344	54.6
**Place of residence**		
Family home	1,936	78.7
Shared flat	157	6.4
Student residence	85	3.5
Alone	284	11.5

### Data Analysis

Descriptive statistics for all variables were computed at an aggregate level as well as by demographics, BMI and level of adherence to the MedDiet (MEDAS score). Adherence to the MedDiet was based on a 14-point scoring system (12) using the same cut-offs (Low: ≤5; Medium: 6–8; High: ≥9) used by Rodríguez-Pérez et al. (5). Depending on the nature of the variable, differences between the segments were evaluated by the Analysis of Variance (ANOVA) or Pearson's Chi-squared (χ^2^) test for count data with Yates continuity correction. Accordingly, Hedge's *g* and Cramer's *V* (13) were used as measures of effect size, respectively, for quantitative and count data.

All analyses were performed in R (14). Statistical significance was set at α = 5%.

## Results and Discussion

According to Table 1, the sample population (*N* = 2,462) was well-distributed in terms of territorial coverage over the Danish regions. A prevalence of women (71.1%) and respondents with higher education was observed (88.5% completed higher education). These figures are virtually identical to those reported by Rodríguez-Pérez et al. (5), and likely reflect the fact that women and university educated respondents are more likely to voluntarily participate in food and health studies. The mean MEDAS score was 5.4 (SD: 2.1, Median: 5, Range: 0–12), about a point lower than that reported for the Spanish population [6.5, (5)], indicating an expected lower adherence to the MedDiet in Denmark compared to Spain. Remarkably, more than 50% of the sample was classified as having a low adherence to MedDiet and only about 7% as having a high MEDAS scores (Table 1—the figures in the Spanish population were 17 and 28%, respectively).

Adherence to MedDiet in the Danish population was slightly higher in females than in males (mean MEDAS score 5.6 vs. 5.1, respectively, Hedges' *g* = 0.25 *p* < 0.001), and in older individuals (65+: 5.9, 51–65: 5.8) compared to younger (36–50: 5.2, 18–35: 5.4; all comparisons between older and younger groups were significant, Tukey *p* ≤ 0.02, albeit effect sizes were small to moderate, Range (*g*) = 0.04–0.32). Furthermore, individuals in the “Low” MEDAS group had a significantly higher mean BMI (25.3 kg/m^2^) than both the “Medium” (24.1, *g* = 0.27, *p* < 0.001) and “High” (23.3, *g* = 0.41, *p* < 0.001) groups, whereas the difference between the Medium and the High groups was smaller (*g* = 0.20), although approaching marginal significance (*p* = 0.096). This suggests, as expected, that a higher adherence to the MedDiet is associated with lower body mass, and was further confirmed by the observed negative correlation between the raw MEDAS score and BMI (*r*_(2, 460)_ = −0.19, *p* < 0.001).

Table 2 shows changes in general dietary habits and physical activities reported by participants as having happened during the lockdown period. A substantial proportion of participants reported having snacked more frequently (41.7%), cooking more (29.9%), and overall having eaten more (42.8%) during the lockdown period (Table 2). These results reflect the fact that people spend a significant amount at home during the lockdown and are consistent with expectations that the stress and boredom associated with the lockdown may have increased emotional eating (5, 7). Importantly, all three figures were significantly higher in women than in men and were significantly lower in participants with a high MedDiet adherence compared to the medium and low groups (Table 2). With respect to physical activity, almost half of the sample (47.7%) reported having exercised less, most likely due to the limitations in place during the lockdown periods (fitness centers were closed, group sports were forbidden, etc.). Furthermore, almost 30% reported gaining weight during the lockdown. An additional 22% reported not being sure about weight change. These figures for the Danish population are consistent with available reports from other countries, notably Spain (5) and Italy (4). Again, results for physical activity and weight gain indicate respondents with medium-low adherence to MedDiet were more negatively affected, as these groups more often reported a decrease in physical activity and a weight gain compared to high MEDAS counterparts. It is also noteworthy, however, that 29% of the sample reported an increase in physical activity during lockdown, a figure that was slightly lower for the low MEDAS group than the other two groups (Table 2). With respect to gender, the results show no significant differences in changes to physical activity between males and females (Table 2). Yet, women still more often reported weight gain (female 30.5%, male 23.3%), and that corresponds to the fact that women also report eating more in general (female 46%, male 35% (Table 2).

**Table 2:** Reported changes (%) in intake of selected product categories during lockdown, at an overall level as well as by gender and adherence to MedDiet.

		**All**	**Gender**^****a****^				**MEDAS group**				
			**Male**	**Female**	**Cramer's V**	**p**	**Low**	**Medium**	**High**	**Cramer's V**	**p**
Snacking	Lower	10.8	8.5	11.8	0.12	* <0.001*	9.7	11.4	15.4	0.06	*0.004*
	As before	47.5	56.9	43.6			45.8	48.5	53.8		
	Higher	41.7	34.6	44.6			44.5	40.1	30.8		
Cooking frequency	Lower	5.6	5.8	5.6	0.07	*0.003*	6.9	5.0	0.6	0.06	* <0.001*
	As before	64.5	69.2	62.5			65.5	63.9	60.4		
	Higher	29.9	25.0	31.9			27.6	31.2	39.1		
Eating more in general	No	57.2	65.0	54.0	0.10	* <0.001*	54.0	59.6	65.7	0.07	*0.002*
	Yes	42.8	35.0	46.0			46.0	40.4	34.3		
Physical activity	Not active	2.3	2.5	2.2	0.04	0.166	3.4	1.0	2.4	0.08	* <0.001*
	Lower	47.7	45.6	48.6			49.8	46.6	39.1		
	As before	21.0	23.7	19.8			20.3	20.3	29.6		
	Higher	29.0	28.1	29.3			26.4	32.1	29.0		
Weight gain	Yes	28.4	23.3	30.5	0.07	* <0.001*	31.6	25.6	21.9	0.07	* <0.001*
	No	49.4	57.3	46.2			45.0	53.0	59.2		
	Don't know	22.2	19.4	23.4			23.4	21.4	18.9		

*Differences between gender and MEDAS groups were assessed by Pearson's χ^2^ test. Associated effect sizes (Cramer's V) and p-values are reported. Significant p-values (<0.05) are italicized*.

a *The four respondents who did not identify as either male or female (cf., Table 1) are not included in this analysis*.

Table 3 displays results pertaining to reported changes in the intake of individual food categories related to the MedDiet patterns. At the aggregate level, the lockdown resulted in a number of major changes (Table 3). The food categories that were most notably affected by the lockdown were a higher intake of both commercial (21.1%) and (especially) homemade pastries (38.1%), and a higher intake of alcohol and carbonated beverages (Table 3). These results appear fully consistent with the expectation of a higher rate of emotional eating during this period (7) and, in the case of homemade pastries, with the earlier result that many participants reported cooking more during lockdown. At the same time, a significant percentage of respondents reported a lower intake of commercial pastries (18.4%), fried foods (17.7%) and fast food (25.4%) which can also be attributed to the longer time spent at home and limitations to the foodservice sector.

**Table 3:** Reported changes (%) in general dietary habits and physical activity level during lockdown, at an overall level as well as by gender and adherence to MedDiet, with associated χ^2^ *p* values.

		**All**	**Gender**^****a****^				**MEDAS group**				
			**Male**	**Female**	**Cramer's V**	**p**	**Low**	**Medium**	**High**	**Cramer's V**	**p**
Olive oil	Lower	5.7	5.2	5.9	0.02	0.680	5.4	6.3	3.6	0.08	* <0.001*
	As before	88.8	89.7	88.5			91.0	87.1	83.4		
	Higher	5.5	5.1	5.7			3.6	6.6	13.0		
Vegetables	Lower	19.5	12.4	22.4	0.11	* <0.001*	21.4	18.4	16.3	0.07	* <0.001*
	As before	69.2	76.4	66.3			69.9	67.6	69.7		
	Higher	11.3	11.2	11.3			8.6	14.0	14.0		
Fruit	Lower	24.9	21.0	26.5	0.08	* <0.001*	27.0	23.6	17.2	0.05	*0.022*
	As before	64.0	70.2	61.5			62.9	64.7	68.0		
	Higher	11.1	8.8	12.0			10.1	11.7	14.8		
Red meat	Lower	12.3	9.9	13.2	0.05	0.071	10.2	14.1	16.0	0.07	* <0.001*
	As before	76.2	78.7	75.3			75.5	76.7	78.7		
	Higher	11.5	11.4	11.5			14.2	9.2	5.3		
Carb beverages	Lower	5.6	6.9	4.9	0.05	*0.028*	5.0	6.3	5.3	0.10	* <0.001*
	As before	73.0	74.3	72.6			68.2	76.8	85.8		
	Higher	21.4	18.8	22.5			26.9	16.9	8.9		
Legumes	Lower	8.6	5.2	9.9	0.08	* <0.001*	9.2	8.4	4.7	0.06	*0.001*
	As before	84.4	88.6	82.7			85.5	83.1	83.4		
	Higher	7.1	6.2	7.3			5.3	8.4	11.8		
Fish	Lower	8.4	6.6	9.1	0.07	*0.003*	7.8	9.5	6.5	0.06	*0.002*
	As before	75.8	80.5	73.9			78.7	73.2	70.4		
	Higher	15.8	12.9	17.0			13.4	17.4	23.1		
Pastries (commercial)	Lower	18.4	15.5	19.5	0.07	*0.001*	16.2	20.5	20.7	0.10	* <0.001*
	As before	60.6	66.1	58.3			57.1	63.7	66.9		
	Higher	21.1	18.4	22.2			26.6	15.8	12.4		
Pastries (homemade)	Lower	8.1	6.9	8.6	0.13	* <0.001*	7.3	8.4	12.4	0.05	*0.007*
	As before	53.8	64.0	49.6			51.8	55.5	58.0		
	Higher	38.1	29.1	41.8			40.9	36.0	29.6		
Fried foods	Lower	17.7	16.5	18.2	0.03	0.291	17.7	18.2	14.8	0.04	0.104
	As before	78.2	78.5	78.1			77.4	78.1	84.0		
	Higher	4.1	4.9	3.8			4.9	3.6	1.2		
Alcohol	Lower	14.1	13.4	14.3	0.05	0.037	13.0	15.1	16.0	0.03	0.178
	As before	55.6	59.5	53.9			57.6	52.9	56.8		
	Higher	30.3	27.1	31.7			29.4	32.0	27.2		
Fast food	Lower	25.4	25.6	25.3	0.01	0.773	24.8	25.9	26.6	0.08	* <0.001*
	As before	59.5	58.6	59.9			56.4	62.1	66.9		
	Higher	15.1	15.8	14.8			18.8	12.0	6.5		

a *The four respondents who did not identify as either male or female (cf. Table 1) are not included in this analysis*.

*Differences between gender and MEDAS groups were assessed by Pearson's χ^2^ test. Associated effect sizes (Cramer's V) and p-values are reported. Significant p-values (<0.05) are italicized*.

Other notable changes include a lower intake of fruit and vegetables reported by, respectively, 24.9 and 19.5% of participants. A possible contributor to this is that many companies in Denmark provide fresh fruit for their employees, and therefore it could be expected that for some participants, not being physically at work may have resulted in lower fruit consumption. Similarly, a substantial fraction of the consumption of vegetables is in relation to lunch and dinner, and the patterns for their preparation has been substantially changed during lockdown (see below). Interviews in media and reports from Danish retailers in the lockdown period and following show some of these large changes as well. The Danish retail chain Coop (market share around one-third of Danish retail sales), briefly reports an analysis of the changes in the sales of different food categories in the first weeks of the lockdown [measured in Week 13 + 14, 23 March to 5 April (15)]. They report a 97 and 83% growth in sales of flour and yeast, respectively, compared to same time in 2019. Across the retail sector, Nielsen Retail analysis reports an increase of 63% in the sales of flour in March and April 2020 (16). The sales of sweetened beverages (carbonated and un-carbonated pooled in the analysis), had an increase of 10% compared to 2019 in weeks 13 and 14 compared to the same weeks in 2019 (15). All this is consistent with the reported changes we observed. Madkulturen (a self-governing knowledge and change agency under Danish Ministry for Environment and Food), regularly carries out and reports food consumption among Danish citizens. In May 2020 they issued a brief special report regarding changes in food culture as a consequence of the lockdown (17). It revealed that 36% of Danes to some, high or very high degree have seen their food habits affected by the lockdown. The most notable changes that correspond to the present results are that 26% report eating more of “unhealthy foods,” while 9% report eating less of them. In addition, 16% report increase in drinking alcoholic beverages (wine, beer or other) with their dinner, while 9% report decreases. For many of those, the reported changes are related to increases in the consumption of snacks, other in-between meals and alcohol.

Differences between genders did not appear major, as indicated by the relatively small effect sizes^1^ (Cramer's V range: 0.02–0.13), although they were in most cases statistically significant due to the large sample size. The clearest difference between genders pertained to the consumption of pastries (both commercial and homemade) which increased significantly more for women than for men (Table 3). This mirrors the findings by Madkulturen, where 32% of women report eating more of unhealthy foods, and of snacks, while 19% of men report this (17). With respect to MEDAS groups, Table 3 indicates that as for gender, differences between the groups were not very large (Cramer's V range: 0.03–0.10), and overall a majority of participants reported no change in intake for most food categories. However, for those participants that did report changes, the direction of the differences suggests that the participants in the High group increased intake in food categories positively associated with the MEDAS score (e.g., olive oil, fruit, fish) and decreased intake in items negatively associated with it (e.g., red meat, carbonated beverages, commercial pastries), whereas the low MEDAS group showed the exact opposite trend (Table 3). Again, this is in correspondence with Madkulturen's findings (17). They report, that among those that report their food habits affected by the lockdown, 40% perceive the changes overall as positive (e.g., eating healthier), whereas 29% report them as negative (e.g., eat less healthily, eat more take-away).

Taken collectively, the results indicate a pattern whereby individuals with less healthy dietary habits (Low MEDAS) were affected negatively (weight gain, lower physical activity, etc.) by the lockdown, whereas individuals with initial healthier eating patterns (High MEDAS) moved in a healthier direction characterized by increased physical activity and an even higher-level adherence to the MedDiet.

This study confirms some, but not all, of the earlier findings reported in the COVIDiet International project concerning the Spanish population (5). Findings that could be replicated in both countries were that individuals with a high MedDiet adherence reported an increased intake of MEDAS related food categories during lockdown, although this was not true for all food categories (for example, intake of pastries generally increased even for high MEDAS Danes whereas in Spain it decreased). The major difference pertains to the MEDAS score, which was lower in the Danish population. This is of course not entirely surprising since the dietary habits in a Mediterranean country should indeed be closer to the MedDiet ideal than in a Nordic country like Denmark. The main conclusion from the Spanish study was that adherence to the MedDiet increased in the Spanish population during the lockdown (5). The results for the Danish cohort suggest that this was the case only for people characterized by a high MEDAS score, whereas participants with a low adherence to MedDiet (over 50% of the sample) were more negatively affected by the lockdown.

Limitations of this study are discussed in depth in Rodríguez-Pérez et al. (5). We briefly acknowledge here the convenience sampling strategy, which resulted in the overrepresentation of certain categories, particularly women and people with higher education. This was also the case in all countries participating in the COVIDiet international project and reflect the fact that these categories are more likely to be interested in food and health topics and therefore more likely to participate in such studies. Moreover, while the results are based on a large sample size and employed empirically supported measures like the MEDAS score, participants' accuracy in reporting their dietary habits before and during the lockdown is uncertain given that the correlation between self-reported and actual intake can be assumed to be positive, but imperfect (18). Future studies, which are strongly advised to assess the *long-term* effects of the lockdown on dietary habits, can productively address both limitations by employing quota-sampling and a more accurate dietary assessment methods such as 3-day dietary recall or diet history interviews.

## Conclusions

This brief paper focused on changes in dietary habits during the COVID-19 lockdown in the Danish population. The data presented in the paper, based on a self-administered web-based questionnaire (*N* = 2,462), suggest that the lockdown has affected lifestyle and dietary habits of some adult Danes. Main findings include a substantial proportion of participants who reported eating more, snacking more, and exercising less, and consequently gaining weight during the lockdown. It corresponds to other reports of changes in dietary patterns during the lockdown in Denmark (17), as well as the observed changes in purchase patterns in retail (15). Many of the results presented could be linked to the amount of time spent at home (e.g., a higher cooking frequency), a higher degree of emotional eating during the lockdown (e.g., a higher consumption of pastries and alcohol), and generally showed that women were affected to a higher degree than men. Additionally, dietary changes during the lockdown to a certain degree reflected pre-existing (un)healthy eating habits as measured by adherence to the Mediterranean Diet, where positive health outcomes were observed in participants with a high MEDAS score and negative outcomes (e.g., weight gain and higher intakes of pastries and carbonated beverages) were associated with participants with a low MEDAS score. These changes, if sustained long-term are potentially concerning from a public health perspective, especially given that more than half of the participants were characterized by a low adherence to the MedDiet.

## AGR-141 Research Group

Esther Molina-Montes, Vito Verardo, Reyes Artacho, Belén García-Villanova, Eduardo Jesús Guerra-Hernández and Maria Dolores Ruíz-López. Department of Nutrition and Food Science, University of Granada, Campus of Cartuja, Granada, Spain.

## Data Availability Statement

Raw, anonymized data can be obtained upon reasonable request to the corresponding author and after approval by the COVIDiet project coordinator. Requests to access the datasets should be directed to Davide Giacalone, dg@iti.sdu.dk.

## Ethics Statement

The studies involving human participants were reviewed and approved by Research Ethics Committee of the University of Granada. The patients/participants provided their written informed consent to participate in this study.

## Author Contributions

DG: conceptualization, investigation, visualization, formal analysis, writing—original draft, writing—review and editing. MF: conceptualization, investigation, writing—review and editing. CR-P: conceptualization, methodology, data curation, project administration, writing—review and editing. All authors contributed to the article and approved the submitted version.

## Conflict of Interest

The authors declare that the research was conducted in the absence of any commercial or financial relationships that could be construed as a potential conflict of interest.

## Footnotes

^1^With 3 d.f.—which is the case for all comparisons in Table 3—Cohen (13) suggested the following rule of thumb for interpretating Cramer's V values: 0.06 = small effect, 0.17 = medium effect, 0.29 = large effect.
